# Online Testing Method for the Fine Spectral Characteristics of Narrow-Band Interference Filters Based on a Narrow-Linewidth Tunable Laser

**DOI:** 10.3390/s24041152

**Published:** 2024-02-09

**Authors:** Kaijun Ji, Yong Yang, Xin Lin, Jiaming Liang, Kaijie Ji, Jiqin Wang, Linmei Liu, Zhenwei Chen, Wei Wang, Xuewu Cheng, Faquan Li

**Affiliations:** 1 Innovation Academy for Precision Measurement Science and Technology, Chinese Academy of Sciences, Wuhan 430071, China; jikaijun16@mails.ucas.ac.cn (K.J.); linxin@apm.ac.cn (X.L.); liangjiaming@apm.ac.cn (J.L.); jikaijie22@mails.ucas.ac.cn (K.J.); wangjiqin18@mails.ucas.ac.cn (J.W.); liulinmei@apm.ac.cn (L.L.); chenzhenwei@apm.ac.cn (Z.C.); wangwei@apm.ac.cn (W.W.); lidar@apm.ac.cn (X.C.); lifaquan@apm.ac.cn (F.L.); 2 University of Chinese Academy of Sciences, Beijing 100049, China

**Keywords:** narrow-band interference filters, tunable laser, transmission spectrum

## Abstract

The transmission spectrum of a narrow-band interference filter is crucial and highly influenced by factors such as the temperature and angle, thus requiring precise and online measurements. The traditional method of measuring the transmission spectrum of an interference filter involves the use of a spectrometer, but the accuracy of this method is limited. Moreover, placing a narrow-band interference filter inside a spectrometer hinders real-time online measurements. To address this issue, there is demand for high-precision online spectral testing methods. In response to this demand, we propose and experimentally validate a fine spectral characterization method for narrow-band interference filters. This method uses a narrow-linewidth tunable laser, achieving a spectral resolution in the MHz range for online testing. Two types of narrow-band interference filters were tested using the constructed laser spectroscopy experimental system, obtaining a transmission spectrum with a spectral resolution of 318 MHz. In comparison to spectrometer-based methods, our proposed method demonstrates higher spectral accuracy, enables online measurements, and provides more accurate measurements for special spectral interference filters. This approach has significant application value and promising development prospects.

## 1. Introduction

An interference filter is an essential device designed to eliminate background light, thereby enhancing the signal-to-noise ratio of photodetectors. An interference filter utilizes a thin-film interference mechanism to separate the signal spectrum from unwanted spectra [1,2,3]. The primary parameters used to characterize the performance of an interference filter are associated with its transmission spectrum. These parameters include the central wavelength, full width at half maximum (FWHM), out-of-band suppression ratio, and transmission function.

Interference filters, being compact optical devices, are commonly encapsulated within large photodetection systems. For instance, in the collimation optics of the Solar Dynamics Observatory (SDO) [4], various interference filters with different central wavelengths are installed to achieve imaging observations of the Sun in different spectral bands. In atmospheric detection lidar systems, interference filters are incorporated into the weak-signal-detection optical path to separate, filter, and return the signals in different spectral bands. In these applications, the precise measurement and control of the central wavelength and FWHM of the interference filters are crucial to meet the detection requirements of the systems.

As the demand for high and ultra-high spectral resolution detection persists, the transmission function of narrow-band interference filters becomes increasingly important. For example, MASP [5] leverages the characteristic of the interference filter transmission function, changing with the angle to separate the spectral lines at different rotational levels of O_2_ molecules, thus enabling the measurement of Boltzmann temperature. The accurate measurement and calculation of the transmission function of interference filters and their variation at different angles are essential steps in the temperature inversion process, particularly in applications like MASP, where the separation of spectral lines is critical for precise temperature measurements.

The transmission function of interference filters is highly sensitive to the angle. This sensitivity arises because, when the angle of incident light changes, the path length of light passing through the interference film changes accordingly. As a result, the internal interference affects change, leading to corresponding variations in the central wavelength, FWHM, and peak transmittance of the transmission spectrum [6]. Similarly, when divergent light passes through an interference filter, only the central light ray is perpendicular to the surface, while all other rays have certain angular offsets, causing changes in the transmission function [7]. Additionally, factors such as the thermal expansion of coating materials and substrates, as well as the temperature dependence of refractive indices, contribute to the sensitivity of interference filter transmission functions to environmental temperature changes. The central wavelength varies linearly with the environmental temperature, shifting towards longer wavelengths as the temperature increases and towards shorter wavelengths as the temperature decreases. The shift in the central wavelength at longer wavelengths can reach up to 0.03 nm/K [7]. Since conditions such as the temperature and angle during measurements cannot be perfectly aligned in real-world applications, there is a practical need for online measurements of the transmission spectrum of interference filters.

The transmission spectrum of interference filters is typically measured using a spectrometer [2,8,9,10,11]. A spectrometer utilizes the principle of optical dispersion to separate the light signals into different components based on the wavelength. It is used to measure the intensity of light at each wavelength point, enabling spectral analysis. Spectrometers use devices, such as xenon or mercury lamps, to provide continuous broadband spectra. Dispersion elements, like gratings, diffraction gratings, or prisms, are employed to disperse the broadband spectrum, and a scanning slit selectively illuminates different pixel points on a charge-coupled device (CCD) array. The entrance slit controls the diameter and angle of the incident beam entering the spectrometer, while the exit slit selects a specific wavelength range for measurement. Due to practical manufacturing limitations, both the entrance and exit slits cannot be infinitely small, leading to the instrumental broadening of spectrometers. This inherent broadening limits the spectral resolution of the spectrometer.

When measuring a working interference filter with a spectrometer, the process typically involves removing the interference filter from its operational setup and placing it in the sample holder of the spectrometer. Once the incident light enters the spectrometer through the entrance slit, dispersion elements (such as gratings or prisms) disperse the incident light. The dispersed quasi-monochromatic light passes vertically through the interference filter and exits through the exit slit. The transmitted light is then received by a detection element and converted into an electrical signal. By scanning the exit slit, the signal intensity of transmitted light is measured at various wavelengths, ultimately producing the transmission spectrum. After the measurement has been completed, the interference filter is placed back into the application system. However, temperature and angle differences in the measurement setup of the spectrometer and the actual optical path of the application system can lead to disparities between the measured and real-world transmission spectra. Adjustments to the angle of the interference filter may be necessary once it has been placed back into the application system. This limitation means that these spectrometers cannot achieve online measurements of the transmission spectrum of interference filters. The current spectral resolution of the spectrometer can reach approximately 0.01 nm, while developing a higher-precision spectrometer would require huge investment.

With increasing demand for a high spectral resolution and advancements in filter technology, interference filters with very narrow transmission bandwidths have gained significant attention. These filters, known as narrow-band interference filters, have found applications in pure rotational Raman lidar [12,13,14,15], astronomical narrow-band imaging observations [16,17,18,19], and other fields. The transmission function of narrow-band interference filters directly influences the accuracy and precision of the data inversion results. Therefore, filters with even narrower bandwidths place more demand on the spectral resolution of the testing methods for transmission spectra. Sensitivity to the temperature and angle in the transmission function highlights the need for online measurements in testing methods. Currently, there is a lack of online measurement methods with a high spectral resolution.

Since the invention of the laser, laser spectroscopy has enabled high-precision spectral measurements over narrow ranges. For instance, in references [20,21], laser spectroscopy techniques were applied to atomic spectroscopy measurements, achieving hyperfine atomic spectra with resolutions reaching the MHz level. With the development of tunable laser technology, infrared wavelength lasers with a wide tuning range (in the order of nanometers) have become readily available [22,23,24]. The application of tunable infrared lasers in dense wavelength division multiplexing (DWDM) systems has greatly increased the number of communication channels [25], and the minimum wavelength spacing between two bands is as small as 0.2 nm. The narrow-band interference filter, which is used to isolate different wavelength signals, plays a crucial role in DWDM. To improve the success rate and obtain higher-quality filters, a tunable laser is introduced to precisely control and optimize the deposition process during filter manufacturing [26].

In recent years, tunable technology in the visible wavelength range has made significant progress [27,28,29,30]. The emergence of narrow-linewidth and widely tunable, visible lasers has provided the possibility of using laser spectroscopy for the precise measurement of the transmission spectra of narrow-band interference filters in the visible wavelength range. In this study, we propose and experimentally implement a new method for online measurement of the transmission spectrum of narrow-band interference filters using laser scanning, achieving high spectral precision. The spectral resolution can reach the MHz level, providing a novel approach for the online detection of narrow-band interference filters, birefringent filters, and other high spectral resolution instruments.

## 2. Experiment Principles and Plan

In order to achieve high spectral precision in measuring the transmission spectrum of interference filters, a new method utilizing laser scanning is proposed. The laser scanning method utilizes the principles of laser spectroscopy to measure the transmission spectrum. It employs a laser with a narrow linewidth and broad tunability as the light source and a point detector to capture the intensity of the light passing through the narrow-band interference filter. The broadening of instruments in laser scanning is primarily constrained by the linewidth of the laser. The currently available tunable lasers can achieve linewidths in the order of 100 kHz, allowing users to easily achieve high spectral resolution measurements.

**Figure 1:**
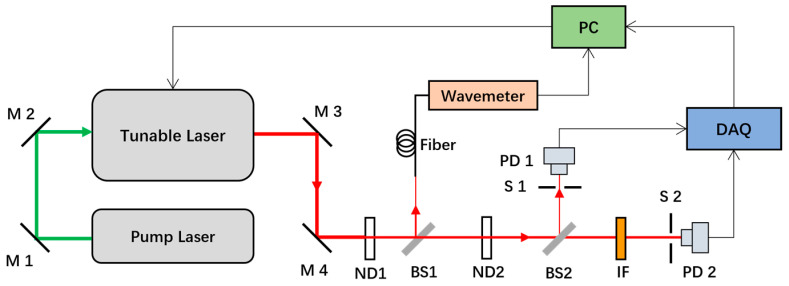
Schematic of the experiment. M1–M4: mirror; ND1 and ND2: neutral density; BS1 and BS2: beam splitter; S1 and S2: shutter; IF: interference filter; PD1 and PD2: photo diode detector; DAQ: data acquisition card; PC: computer.

The experimental principle of the laser scanning method is illustrated in Figure 1. The laser was split into two beams by a beam splitter (BS1) after passing through the neutral density filter (ND1). Among this, 1% was reflected into the receiving fiber of the wavemeter and the laser wavelength was measured by the wavelength meter. Meanwhile, 99% of the laser light passed through the beam splitter (BS1), and the power was adjusted by the neutral density filter (ND2). Following this procedure, the laser light was also divided into two beams by the beam splitter (BS2), of which 1% of the laser light was reflected into the photodetector (PD1) for laser power monitoring. The remaining 99% passed through the beam splitter (BS2) and was vertically incident on the interference filter. The transmitted light entered the photodetector (PD2) for the measurement of the signal intensity of the interference filter. The photodetectors (PD1 and PD2) converted the light intensity signal into a voltage signal, which was acquired by the data acquisition card (DAQ). The wavelength data measured by the wavemeter, the two voltages collected by the data acquisition card, and the time information were recorded synchronously by running the self-written real-time processing and display program on a computer. The main parameters of the devices used in the experiment are detailed in Table 1.

The linewidth of the laser affects the spectral resolution, thereby causing the laser to have a narrow-linewidth. The laser’s output wavelength needs to cover the transmission band of the interference filter, necessitating a tunable laser with a wide tuning range. The laser light source used in the experiment was generated by a Matisse C continuous ring cavity Ti:sapphire laser from Spectra-Physics Company (Milpitas, CA, USA). When Matisse C is in operation, it is pumped by a laser with an output wavelength of 532 nm (Millennia EV). The wavelength coverage range of Matisse C is 700–1000 nm, and the linewidth is less than or equal to 100 kHz. A Matisse C laser can achieve high-precision continuous wavelength scanning through computer control, and its mode-hop-free tuning range is up to 150 pm. Therefore, the laser needs to be reset after scanning up to 150 pm in order to perform the next cycle. The scanning frequency of the Matisse C laser can be manually set. Frequent scanning takes a short time and causes less variation in the environment, leading to a low spectral resolution. In consideration of the experiment duration, spectral resolution, and laser status, the scanning frequency was set to 300 MHz/s.

**Figure 2:**
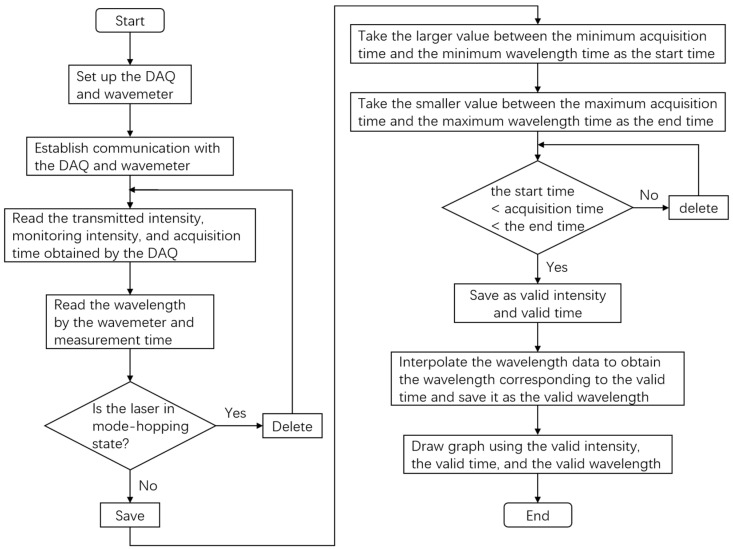
The flowchart of the data processing program.

Additionally, lasers with a large dynamic tuning range may exhibit non-linear scanning speed issues, introducing discrepancies between the transmitted light intensity and time waveform and the actual transmission spectrum. To address this, real-time measurement of the laser wavelength using a wavelength meter is essential. To achieve the synchronous measurement of the laser wavelength and signal intensity, a real-time processing and display program for the laser spectrum data was developed. The flowchart of the program is shown in Figure 2. Using the program, the DAQ was configured to read the optical intensity measured by the PD and record the acquisition time. At the same time, the wavelength measured by the wavemeter and its corresponding time were also recorded. Then, the mode hopping data were eliminated, and interpolation was used to obtain the wavelength corresponding to the acquisition time. This enabled the quasi-simultaneous measurement of the wavelength and optical intensity.

## 3. Test and Results

To assess the universality of the laser scanning method, two narrow-band interference filters with different transmission spectra from different manufacturers were selected for testing: Andover-13804 and Alluxa-7270. The transmission spectra of these filters, provided by their respective manufacturers, reveal distinct characteristics. According to the transmission spectrum measurements taken by using the spectrometers provided by the two manufacturers, it was observed that Andover-13804 exhibits a typical Gaussian-shaped transmission spectrum, while Alluxa-7270 shows a distinct hump-shaped transmission spectrum.

The laser scanning experimental system was placed in a clean laboratory with constant temperature (23 °C) and humidity. First, we evaluated the narrow-band interference filter from Alluxa-7270 using our system. The original data package stored by the signal acquisition program contained the following information: time, wavelength, and two optical intensity channels. To record the intensity variation of the incident light, the light from one channel was blocked from passing through the interference filter. To record the change in the intensity of the transmitted light as the wavelength of the incident light changed, the light from another channel was transmitted through the interference filter.

**Figure 3:**
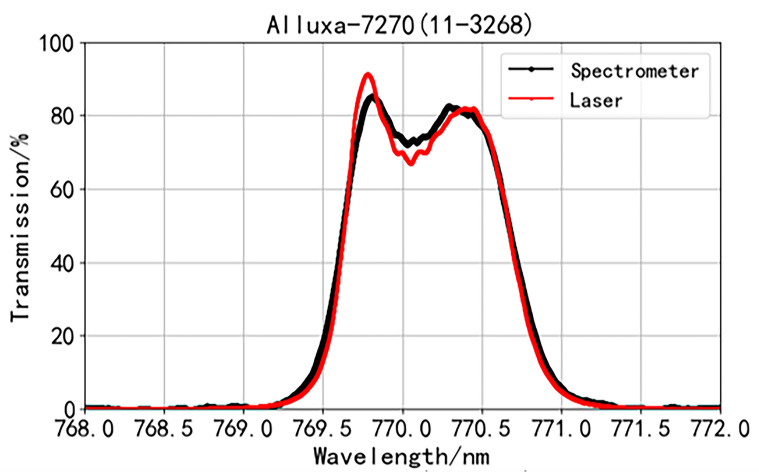
Transmittance function of Alluxa-7270 measured with a scanning laser (red line) and a spectrometer (black line).

In Figure 3, the black line represents the transmission spectrum of the interference filter Alluxa-7270, as measured by the manufacturer using a spectrometer. From this, it can be inferred that the central wavelength of the Alluxa-7270 transmission spectrum is 770 nm, with a transmission bandwidth of 1 nm. Therefore, we chose the laser scanning range from 768 nm to 772 nm. We applied the ratio of the transmitted light intensity to the incident light intensity at the same wavelength as the relative transmittance of the interference filter and then sorted the transmittance by wavelength to obtain the preliminary transmission spectrum results.

During the entire scanning process, the laser experienced mode hopping. Hence, the mode hopping data had to be excluded. Additionally, the collection of light intensity and wavelength is independent and asynchronous, so data processing was required. To determine the valid time period, we selected the maximum value between the minimum collection times and wavelength times as the start time, and the minimum value between the maximum collection times and wavelength times as the end time. Interpolation of wavelength data packets (wavelength and time) was performed based on the valid time period to obtain wavelengths that are synchronized with intensities. Next, we sorted the synchronized data packets (transmission, monitoring intensity, and wavelength) based on wavelength. By calculating the ratio of the transmission to monitoring intensities at each wavelength, we obtained an initial transmission spectrum. However, since the wavelength spacing was uneven at this point, we performed a uniform sampling of the initial transmission spectrum based on the wavelength, resulting in a transmission spectrum with evenly changing wavelengths. The transmission spectrum of the narrow-band interference filter from Alluxa-7270 was obtained from the processed data (Figure 3, red line). According to the processed transmission spectrum, the wavelength interval is 0.629 pm, resulting in an actual spectral resolution of 318 MHz (@ 768–772 nm).

A “camel hump” figure can be observed in the transmission spectrum of the narrow-band interference filter from Alluxa-7270, with a peak on each side of the central wavelength at 770 nm. This finding indicates that the transmittance does not reach the maximum at the central wavelength and, instead, continuously increases from the wavelength of 769.0 nm and reaches a maximum of 90.94% at a wavelength of 769.78 nm. With the increasing wavelength, the transmittance starts to decrease and reaches the local minimum of 66.54% at a wavelength of 770.05 nm. With a further increase in the wavelength, the transmittance rises again, reaches the second peak of 81.67% at a wavelength of 770.39 nm, and finally decreases to zero. A large difference in transmittance is observed between the peaks and the trough: the transmittance at the trough at the central wavelength is 73.17% and 81.47% of the transmittance at the two peaks.

**Figure 4:**
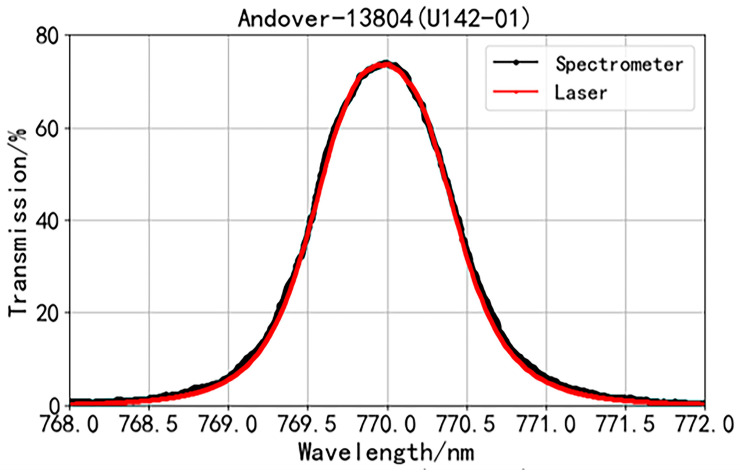
Transmittance function of Andover-13804 measured with a scanning laser (red line) and a spectrometer (black line).

We obtained the transmittance spectrum of the narrow-band interference filter from Andover with the same approach (Figure 4, red line) and found that it is a typical transmittance spectrum for narrow-band interference filters. The transmittance reaches the maximum of 73.58% at a central wavelength of 769.98 nm and then decreases with the increase in wavelength.

## 4. Discussion

**Figure 5:**
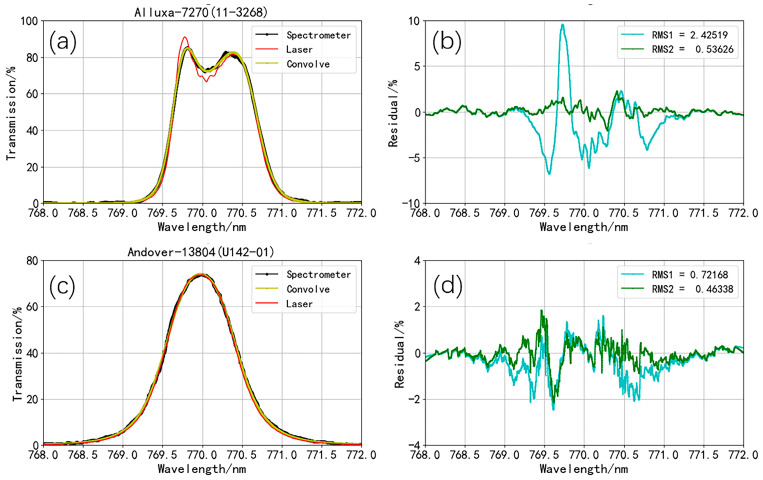
(**a**) After a certain instrument broadening is introduced into the scanning laser-measured result of Alluxa-7270, the consistency with the spectrometer-measured transmission spectrum is higher. (**b**) The difference between the scanning laser-measured result of Alluxa-7270 convolved with an instrument broadening function and the spectrometer-measured transmission spectrum is significantly smaller. (**c**) After a certain instrument broadening is introduced into the scanning laser-measured results of Andover-13804, the consistency with the spectrometer-measured transmission spectrum is higher. (**d**) The difference between the scanning laser-measured result of Andover-13804 convolved with an instrument broadening function and the spectrometer-measured transmission spectrum is smaller.

Comparing our results (Figure 3, red line) with the spectrometer-measured transmission spectrum of Alluxa-7270 provided by the manufacturer (Figure 3, black line), we observed that the waveforms are consistent, as both exhibit a hump shape. However, the laser-scanned transmission spectrum of Alluxa-7270 exhibits steeper inclines and declines in the regions of increasing and decreasing transmittance at the wings compared to those of the spectrometer-measured spectrum. These differences are particularly pronounced in the central transmittance region. The transmittance at the two peaks (769.81 and 770.29 nm) in the spectrometer-measured spectrum are 85.17% and 82.6%, respectively, and the transmittance at the trough (770.03 nm) is 72.06%. The transmittance at the trough is 84.6% and 87.24% of the transmittance at the two respective peaks. The difference in transmittance obtained via the two methods is illustrated by the blue line in Figure 5b, with the maximum difference occurring at 769.72 nm, reaching 9.6%.

The spectrometer-measured transmission spectrum has a resolution of 0.02 nm, whereas the laser-scanned transmission spectrum has a spectral resolution of 0.629 pm. Our system offers around 30-fold higher accuracy of the transmission spectrum compared with that of the spectrometers. Owing to the high spectral resolution, the transmission spectrum obtained by our system presents many details that are not seen in the transmittance spectrum generated by the spectrometer at the trough.

For the transmittance spectrum of the narrow-band interference filter from Andover, the two systems offer almost the same curve (Figure 4). The maximum difference in transmittance is about 2.5% (Figure 5d, blue line). From Figure 4, it can be observed that in the regions where the transmittance increases and decreases on both sides, the laser-scanned transmission spectrum is steeper compared to the spectrometer-measured transmission spectrum, which is consistent with the experimental results for Alluxa-7270.

The spectrometer is limited by the spectral capabilities of its dispersive elements, preventing the complete separation of light at different wavelengths. Simultaneously, the actual aperture widths of the entrance and exit slits cannot be ignored, causing diffraction as light passes through. This results in the instrumental broadening of the obtained transmission spectrum when measuring the interference filters using a spectrometer [31].

Assuming that the real transmission line function of the interference filter is *F*(*λ*), and *λ* represents the wavelength, the instrument broadening function of the spectrometer is *G*(*λ*). Finally, the transmission line *H*(*λ*) obtained with the spectrometer can be described using Equation (1).

(1) $$\begin{array}{l} H\left(\lambda \right)=G\left(\lambda \right)*F(\lambda )\\ \;\;=\int_{-\infty }^{\infty }G\left(\lambda -\lambda^{\prime }\right)F(\lambda^{\prime })d\lambda^{\prime }, \end{array}$$

The discrete form of Equation (1) can be expressed as

(2) $$H\left(\lambda \right)=\sum_{\lambda^{\prime }=-\infty }^{\infty }G\left(\lambda -\lambda^{\prime }\right)F(\lambda^{\prime }),$$

We assumed that the line shape of the instrument broadening function of the spectrometer is Gaussian. The instrument broadening function can be expressed as follows:

(3) $$G(\lambda )=e^{-\frac{\lambda^{2}}{2\sigma^{2}}},$$

where *σ* is the standard deviation and *σ* is given by

(4) $$\sigma =\frac{FWHM}{2\sqrt{2ln2}},$$

where *FWHM* is the full width at half maximum (nm).

The transmission spectrum gained through laser scanning (Figure 5a, red line) is used as the input function *F*(*λ*) of the spectrometer. *F*(*λ*) and *G*(*λ*) (the *FWHM* of G(*λ*) is taken as 0.19 nm) are substituted into Equation (2) to perform a convolution calculation, and the results are shown in Figure 5a (yellow line). We compared the convolution result with transmission spectra generated by the scanning laser and spectrometer and confirmed that the convolution result is relatively consistent with the transmission spectrum measured with the spectrometer (Figure 5a).

For the Alluxa-7270 filter, the maximum value of the transmittance difference (Figure 5b, blue line) is 9.6% before convolution, and the root mean square of the difference is 2.425%. After convolution, the maximum value of the transmittance difference decreases (Figure 5b, green line) to 2.3% and the root mean square decreases to 0.536%.

For the Andover-13804 filter, its trend is similar to that of the Alluxa-7270 filter. The convolution is consistent with the transmission spectrum measured with the spectrometer (Figure 5c). After convolution, the *FWHM* of *G*(*λ*) is taken as 0.19 nm, and the root mean square of the difference decreases from 0.722% to 0.463% (Figure 5d).

Our data reveal that the difference in the transmission spectrum between the scanning laser and spectrometer is due to the instrument broadening of the spectrometer. This instrument broadening effect makes the spectrometer introduce a certain smoothness and broadening to the real transmission spectrum of the narrow-band interference filter. This effect is not so obvious in conventional transmission spectra, as represented in Figure 4, but is evident in the special transmission spectra, and thus cannot be ignored, as exemplified in Figure 3.

**Figure 6:**
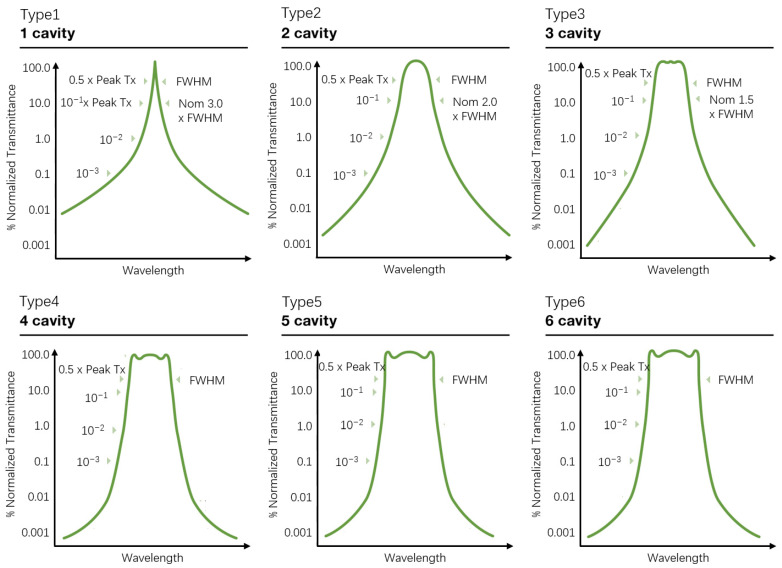
Spectral profiles for Andover’s 6 basic filter types [7].

Andover provided several basic transmission spectrum types of interference filters [7], as shown in Figure 6. In comparison to the Gaussian-type narrow-band interference filters (Types 1 and 2), special-shaped spectra, such as peak-shaped and flat-top, exhibit richer details. Through the comparison of the two testing methods, it can be observed that the laser scanning method has a higher spectral resolution, revealing many details that are not visible with the traditional spectrometer methods. For applications where the transmission function of narrow-band interference filters, such as those in pure rotational Raman lidar and MASP, impacts the accuracy of data inversion, the laser scanning method demonstrates greater practical value. Additionally, for applications involving imaging with narrow-band interference filters, the small laser spot in the laser scanning method allows transmission spectra to be obtained from different regions on the transmission surface by changing the spot’s position. Furthermore, more accurate spectral test results can guide the improvement of simulation theories for the narrow-band interference filter transmission spectra.

## 5. Application

**Figure 7:**
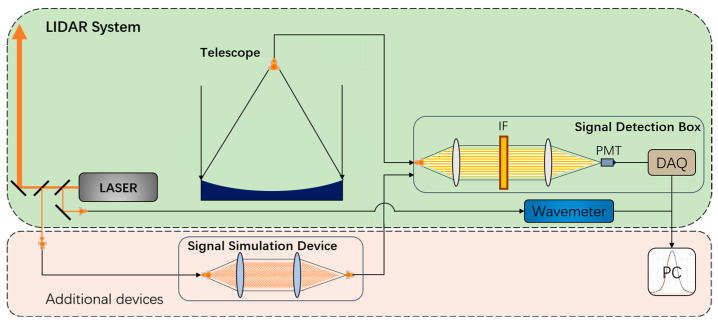
Schematic of the application.

Unlike a spectrometer’s inability to measure the transmission spectrum online, the laser scanning measurement method has broader application prospects in laser spectrum applications due to its unique online measurement capability. Atmospheric detection lidar systems need to operate around the clock, facing factors such as large temperature differences between day and night and inconsistent deformation of mechanical structural components. These factors necessitate online calibration of narrow-band interference filters’ transmission spectra. Figure 7 illustrates an application example of the laser scanning measurement method in an atmospheric detection lidar system. The green area represents some equipment of the atmospheric detection lidar system with the narrow-band interference filter placed in a signal detection box. The pink area includes additional devices, such as the signal simulation device and control software. A small amount of light is extracted from the emitted light of the narrow-linewidth tunable laser belonging to the lidar system, and the signal simulation device adjusts the laser’s divergence angle to simulate lidar echo light. Using the simulated signal light as the source, it is fiber-coupled into the signal detection box, where the narrow-band interference filter is located. Finally, the software controls the operation of the data acquisition card and wavelength meter, providing real-time output and storage of the transmission spectrum of the narrow-band interference filter. The temperature, angle, and other conditions of the narrow-band interference filter remain unchanged before and after the measurement. Moreover, this approach is applicable to various narrow-band filters used in atmospheric detection laser radar systems, such as narrow-band interference filters, Fabry–Perot etalon, and atomic filters. Furthermore, this method can also be extended to the online measurement of filters used in solar observations, such as birefringent filters.

## 6. Conclusions

We constructed a novel system that measures the transmission spectrum of interference filters with high spectral accuracy via laser scanning. We tested our system by detecting the transmission spectrum of narrow-band interference filters and obtained a transmission spectrum with a high spectral resolution. The spectral resolution was able to reach 0.629 pm. Compared with the spectrometer, our system is more accurate and has better online measurement capabilities, providing greater application value in the measurement of special spectral interference filters. In addition, the fine transmission spectrum measured by our system provides guidance for the improvement of the simulation theory of the interference filter transmission spectrum.

## Figures and Tables

**Table 1:** Parameters of the laser scanning experiment system.

Device Component	Parameter	
Tunable Laser	Wavelength range: 700–1000 nm	
	Linewidth: 100 kHz	
Wavemeter	Measurement range: 248–1100 nm	
	Absolute accuracy: 600 MHz	
	Measurement resolution: 20 MHz	
PD	Measurement range: 320–1100 nm	
	Bandwidth: 11 MHz	
	Active Area: 75.4 mm^2^	
DAQ	Channel: 4	
	Measurement range: ±12 V	
	Resolution: 0.0014 mV	
IF	Model: Alluxa-7270	Model: Andover-13804
	Center Wavelength: 770 nm	Center Wavelength: 770 nm
	FWHM: 1 nm	FWHM: 1 nm

## Data Availability

The data presented in this study are available from the corresponding author upon request. The data are not publicly available due to the results of subsequent research not yet being published.
